# Cecal Volvulus in an Elderly Woman: A Rare Cause of Bowel Obstruction

**DOI:** 10.7759/cureus.53571

**Published:** 2024-02-04

**Authors:** Tiago Branco, Leonor Ávila, Ana Rodrigues, Ágata Ferreira

**Affiliations:** 1 General Surgery, Centro Hospitalar do Oeste, Caldas da Rainha, PRT; 2 General Surgery, Centro Hospitalar Lisboa Ocidental - Hospital Egas Moniz, Lisboa, PRT

**Keywords:** emergency abdominal surgery, general surgery, right hemicolectomy, intestinal obstruction, exploratory laparotomy, cecal volvulus

## Abstract

Cecal volvulus is a rare, life-threatening form of bowel obstruction caused by the entanglement of the bowel around the mesenteric axis, compromising blood supply and leading to obstruction and ischemia. The diagnosis is challenging due to its highly variable clinical presentation and differential diagnoses, which may delay timely intervention. This is a case report of an 89-year-old woman who presented with a two-day history of lower right quadrant abdominal pain, nausea, and a temporary loss of consciousness. She also reported a history of chronic constipation. Clinical examination and imaging were suggestive of bowel obstruction, prompting further investigation. Plain radiography and abdominal CT confirmed bowel obstruction, with suspicion of volvulus. The diagnostic uncertainty between cecal and sigmoid volvulus prompted a colonoscopy, which excluded sigmoid volvulus. Emergency laparotomy revealed cecal volvulus and a distended cecum with ischemic changes but without necrosis. A right hemicolectomy was performed, and the patient recovered well postoperatively.

This case report aims to expand the medical knowledge around the topic of cecal volvulus. It underscores the challenges in diagnosing and managing this condition and emphasizes the importance of prompt recognition and surgical intervention to improve patient outcomes.

## Introduction

Volvulus is the twisting of part of the intestine around its mesenteric axis, compromising blood circulation and causing an obstruction in a closed loop, frequently resulting in complete or partial bowel obstruction [1].

Colonic volvulus represents 10% to 15% of all cases of colon obstruction. The most common location of colonic volvulus is the sigmoid colon (80%), followed by the cecum (15%) and the transverse colon (3%). In the context of cecal volvulus, the terminal ileum and the right colon are mostly implicated [2].

Besides congenital or anatomical factors related to a mobile cecum, cecal volvulus is influenced by a range of other risk factors. These include postsurgical adhesions, late-term pregnancy, high-fiber intake, adynamic ileus, chronic constipation, and distant colon obstruction [3-5].

The most common symptoms of volvulus include abdominal pain, constipation, nausea, and vomiting [1-5]. Michele et al. described the patterns of clinical presentation as recurrent intermittent in patients with mobile cecum who typically report chronic, intermittent abdominal pain that spontaneously resolves after passing flatus. On the other hand, those with acute obstruction present with cramping abdominal pain and persistent vomiting that does not resolve spontaneously. Physical examination commonly reveals abdominal tenderness, potentially accompanied by a palpable abdominal mass. In contrast, patients with acute fulminant volvulus often exhibit a toxic appearance along with abdominal tenderness indicative of peritonitis. This severe presentation is frequently associated with bowel necrosis [4]. Signs of cecal volvulus are non-specific and may overlap with other causes of intestinal obstruction. This contributes to the complexity in distinguishing between cecal volvulus and other types of obstruction [1-3,5].

Recognizing the challenges posed by cecal volvulus requires a comprehensive understanding of its clinical intricacies and contributing factors. Clinical suspicion is crucial for identifying cecal volvulus, particularly when patients exhibit symptoms indicative of intestinal obstruction. A delay in diagnosing cecal volvulus can progress to ischemia, necrosis, perforation, and consequent acute peritonitis. The combination of abdominal plain radiography and abdominal computed tomography (CT) enhances diagnostic accuracy. Imaging studies are essential and typically performed to confirm the diagnosis. The effective treatment of cecal volvulus is surgical intervention [1,3].

In this report, we present the case of an 89-year-old female who underwent emergency laparotomy for intestinal obstruction with intraoperative finding of cecal volvulus. Despite her advanced age, she underwent successful and prompt surgical intervention through a right hemicolectomy for cecal volvulus, overcoming initial uncertainties about the specific type of volvulus involved. This case highlights the difficulties and critical importance of prompt diagnosis, as well as the need for proactive management to mitigate the risk of severe complications associated with cecal volvulus.

Informed consent to use clinical information and medical photography was obtained from the patient, and her right to privacy and confidentiality was rigorously upheld throughout this process.

## Case presentation

An 89-year-old woman presented to the emergency department with a chief complaint of a two-day history of continuous and colicky right lower quadrant abdominal pain that was accompanied by nausea. She also reported one isolated episode of temporary loss of consciousness. The patient denied any episodes of vomiting or fever.

She had a history of chronic constipation and colicky abdominal pain for months. The patient's medical history was unremarkable, and she did not take any chronic medication, with the exception of occasional laxatives.

On clinical examination, the patient was hemodynamically stable and apyrexial, with a normal neurological examination. Abdominal examination revealed hypoactive bowel sounds and a distended abdomen with diffuse tenderness mainly in the right lower quadrant. Digital rectal examination was unremarkable, and the remaining systematic clinical examination was normal.

**Figure 1:**
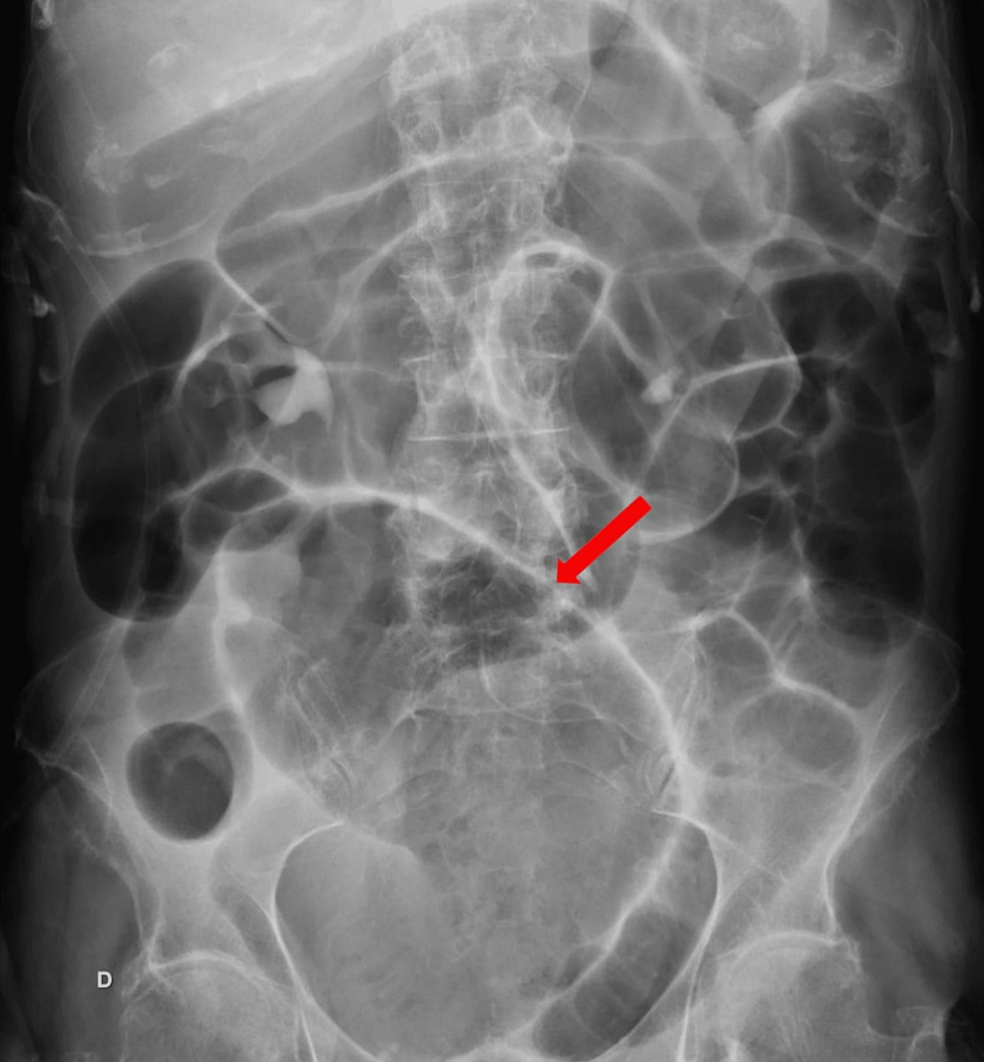
Abdominal X-ray showing the dilated small bowel Red arrow indicates the “coffee bean” sign

Laboratory tests showed evidence of acute renal failure, with a serum creatinine level of 1.22 mg/dL. However, the complete blood count and C-reactive protein levels were within normal ranges. Abdominal plain radiography showed signs of bowel obstruction, including dilated gas-filled small bowel and “coffee bean" sign (Figure 1).

**Figure 2:**
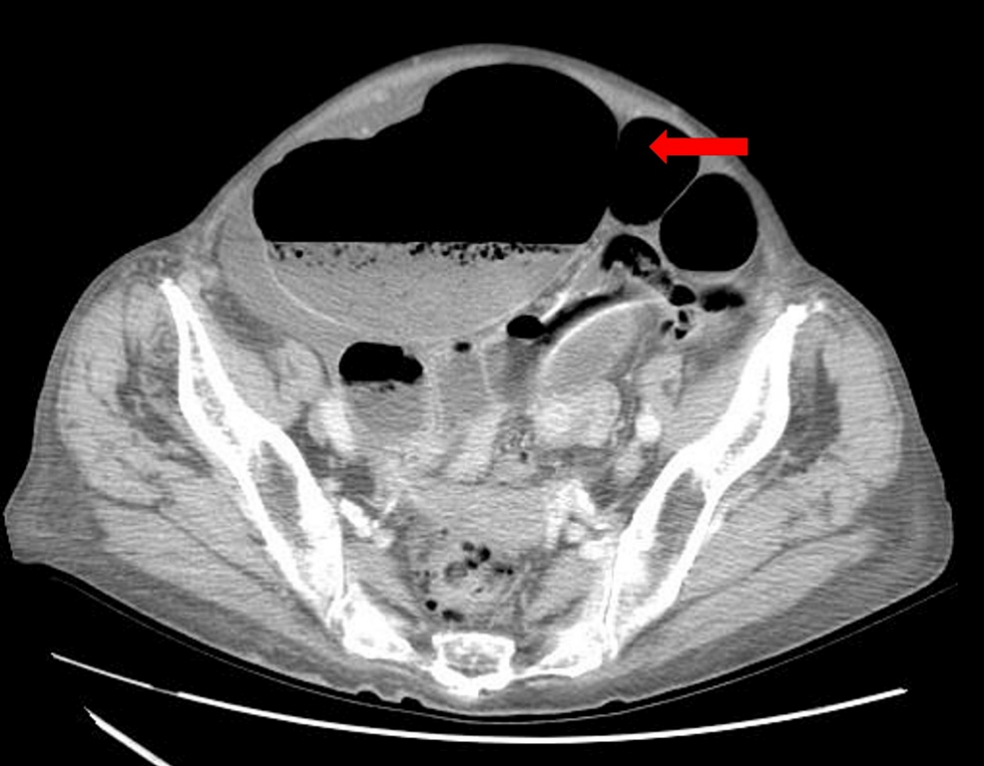
Abdominal CT scan (axial plane) Red arrow indicates a grossly dilated air-fluid distended cecum

**Figure 3:**
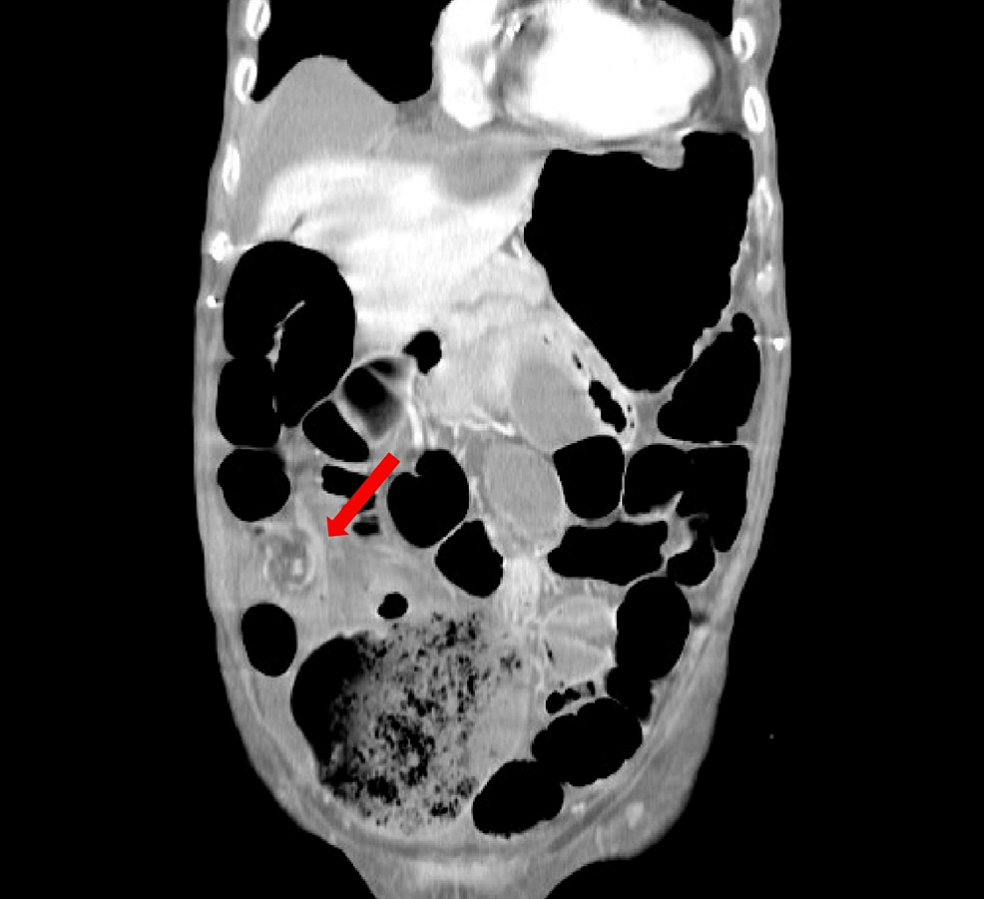
Abdominal CT scan (coronal plane) Red arrow indicates the mesenteric “whirl sign”

Subsequent abdominal CT scan revealed a grossly dilated and distended colon with air and fluid extending to the splenic angle, distended cecum (13.7 cm), and mesenteric “whirl sign” in the right iliac fossa. After consulting the gastroenterology team, a decision was made to perform a colonoscopy to investigate the potential of sigmoid volvulus and assess the feasibility of de-rotation. The colonoscopy ruled out sigmoid volvulus but demonstrated an inability to traverse the ileocecal valve, raising the suspicion of cecal volvulus (Figures 2, 3).

**Figure 4:**
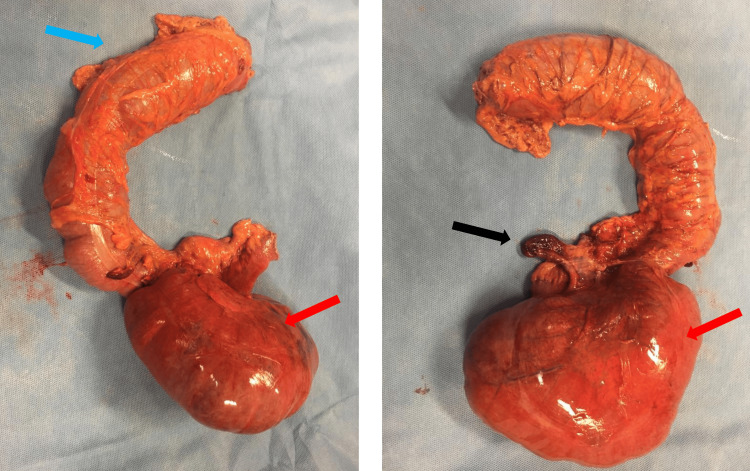
Resected specimen Blue arrow indicates the right colon, red arrows indicate the dilated ischemic cecum, and black arrow indicates the gangrenous appendix

An emergency laparotomy was subsequently performed. Abdominal exploration revealed clear ascitic fluid, small bowel loop dilatation, a distended axial rotated cecum with congestive ischemic wall without necrosis, and a gangrenous non-perforated appendix (Figure 4).

To address the condition, a right hemicolectomy was performed under general anesthesia, involving a side-to-side ileotransverse mechanic anastomosis. The surgical specimen was then sent for histopathological analysis, which revealed distal ileum and cecum changes compatible with ischemic necrosis, intramural edema, areas of steatonecrosis, vascular congestion, and hemorrhage with serositis with no evidence of malignancy. Postoperatively, the patient made a full recovery and was discharged on the eighth postoperative day without any complications reported on the short- and long-term follow-ups.

## Discussion

Cecal volvulus accounts for 1-1.5% of all adult intestinal obstructions [1,2]. It occurs due to the rotation and torsion of the cecum and ascending colon around the mesentery, mainly due to the weak attachment of these structures to the retroperitoneum. [4]. Normal rotation of 270° during intrauterine life with deficient fixation to retroperitoneal structures results in cecocolic mobility, predisposing to cecal volvulus.

Peltrini et al. described three types of cecal volvulus: type 1 or axial torsion type, in which the rotation occurs along the longitudinal cecum-ascendent colon axis, and the location of the cecal volvulus is in the right lower quadrant; type 2 or loop type, where the rotation occurs along the mesenteric axis, and the location of the cecum gets displaced typically to left upper quadrant; and type 3 or cecal bascule type, when the distend cecum folds anteriorly without any torsion [6,7].

Complaints of intestinal obstruction on physical examination, such as abdominal distention, nausea, vomiting, constipation, abdominal tenderness, and/or inability to pass gas or stool, should raise a clinical suspicion of volvulus [1]. Around 50% of patients with cecal volvulus reported to have experienced periodic abdominal pain before [2].

Plain abdominal radiography assists in diagnostic endeavors when demonstrating the non-specific "coffee bean"-shaped cecum in around 25% of patients. This should prompt abdominopelvic CT, which is diagnostic in 90% of the cases. Moreover, the "whirl sign" is pathognomonic for cecal volvulus type I or II. However, around 30% of patients do not demonstrate these radiographic features, and 10% of cases are only diagnosed intraoperatively [1,8-10].

Albeit possible, non-surgical interventions for cecal volvulus have modest results. The success rate for colonoscopic reduction of cecal volvulus is reported to be only 30%, and considering the potential risk of colonic perforation, colonoscopy is not normally recommended in the management of cecal volvulus [11]. Surgery remains the most widely accepted treatment approach [12-14]. There are several surgical options such as right hemicolectomy or cecum resection, followed by ileocolic anastomosis, detorsion and manual reduction of volvulus by celiotomy, cecopexy, fixation of right colon by suturing the cecum and/or ascending colon to lateral parietal peritoneum, and resection of the affected segment if there is extensive intestinal ischemia [4]. All of these may be performed by laparotomic or laparoscopic approaches, depending on surgeon experience. The most effective cecal volvulus approach described in the literature is the laparotomic approach, although laparoscopic cecopexy has recently been described as safe [15,16].

Reported mortality rates range from 10% to 40%, depending on the presence of gangrenous intestine, and medical and surgical approaches [15]. Sigmoid volvulus is more commonly found in older patients, usually men over the age of 70 years, whereas cecal volvulus is more commonly found in women in their second and third decades [1,10].

## Conclusions

Cecal volvulus is an uncommon cause of bowel obstruction and occurs more frequently in younger women. Every suspicion of intestinal obstruction, hinted by the clinical history and physical examination, should prompt radiographic investigation with abdominal plain radiography and abdominal CT. Although non-surgical management strategies have been described, the most effective treatment for cecal volvulus is urgent surgery, which avoids cecal perforation, decreasing perioperative patient morbidity and mortality.
